# Double trouble: a case of bilateral multiple epidermal inclusion cysts after reduction mammaplasty

**DOI:** 10.1080/23320885.2022.2158832

**Published:** 2023-02-17

**Authors:** Khairun Izlinda Abdul Jalil, David Herlihy, Edward Jason Kelly

**Affiliations:** Department of Plastic and Reconstructive Surgery, Cork University Hospital, Cork, Ireland

**Keywords:** Epidermal inclusion cysts, breast surgery, reduction mammaplasty

## Abstract

Epidermal inclusion cysts (EIC) of the breast are uncommon and can occur after previous surgery or trauma. Here we discuss a case of massive bilateral multiple EIC of the breast presenting seven years after reduction mammaplasty. This report highlights the importance of accurate diagnosis and management of this rare condition.

## Introduction

An epidermal inclusion cyst (EIC) is a common cutaneous lesion consisting of keratinizing squamous epithelium within a confined cyst, filled with keratin and lipid rich debris in the dermis or sub-dermis. Such cysts are most commonly found on the face, neck and trunk, but can occur anywhere in the body [1].

EIC in a breast is uncommon. The pathogenesis remains unknown but five theories have been postulated. Firstly, it can develop from obstructed hair follicles [2]. Secondly, it can result from cystic reaction in the dermis from inflamed pilosebaceous structures [3]. Thirdly, it can occur as a result from trauma or surgery [4–7]. Fourthly, EIC of the breast can be congenital [8]. Lastly, it can occur as a result of squamous metaplasia [3].

Here, we present an unusual case of bilateral multiple epidermal inclusion cysts that occurred after reduction mammaplasty for macromastia.

## Case report

**Figure 1.**
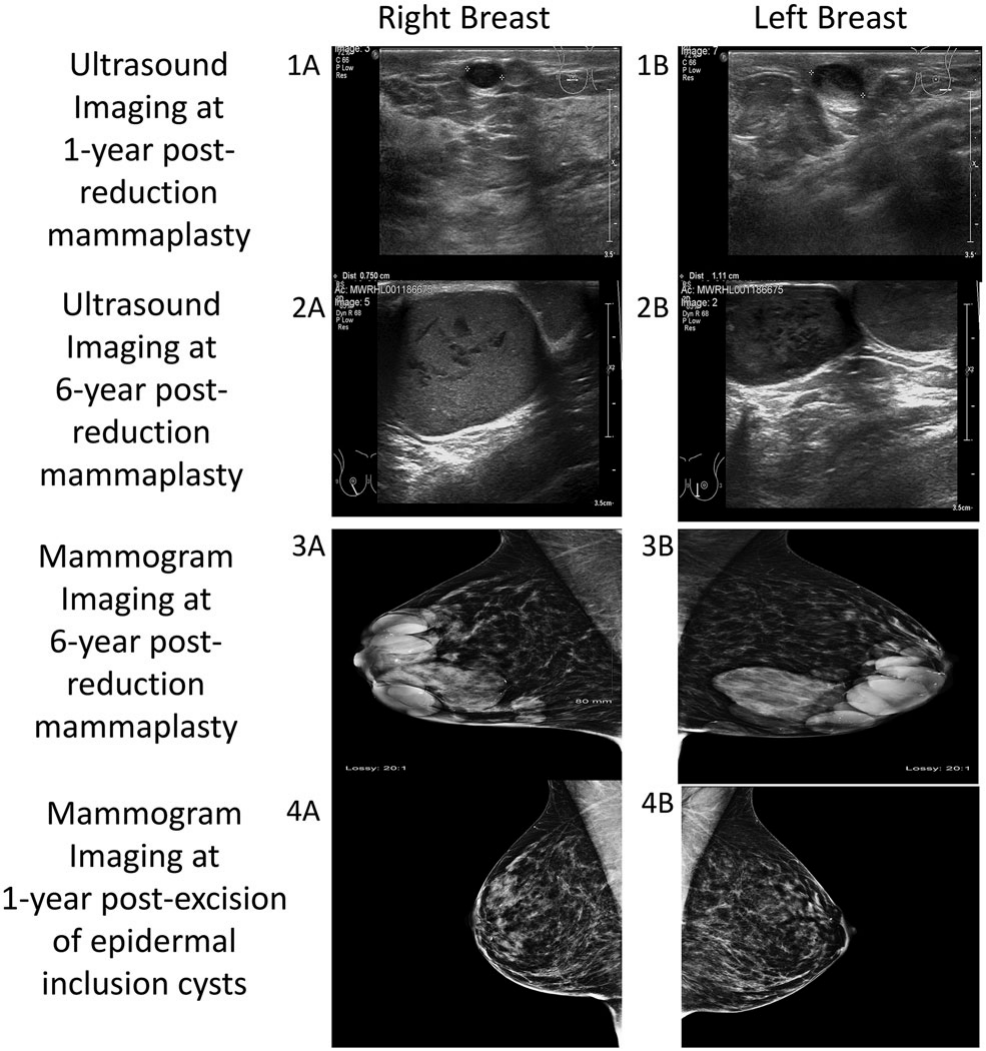
Imaging series showing progression of bilateral epidermal inclusion cysts of the breast. Ultrasound imaging: at one-year post-reduction mammaplasty (1A,1B) and at six-year post-reduction mammaplasty (2A,2B). Mammogram imaging at six-year post-reduction mammaplasty (3A,3B) and at one-year post-excision of epidermal inclusion cysts (4A,4B).

A 38-year-old female presented with bilateral subcutaneous breast masses that were increasing in size. Past medical history was significant only for mild hidradenitis suppurativa (Hurley stage 1) that presented in the axilla and groin. Previous episode of hidradenitis suppurativa flares were successfully managed with topical therapy only and had never required surgical treatment. She was a non-smoker, not on any medication and had a body mass index of 24. Six years prior to this presentation, she underwent a Wise pattern, inferior pedicle type bilateral breast reduction for macromastia. The operation (performed by senior author), included a standard de-epithelialization to create the dermoglandular pedicle, using a scalpel. This was her first surgical procedure. She received one intraoperative dose of antibiotic given and her postoperative course was uneventful. Her bilateral breast scars healed without complication. She was pleased with the cosmetic outcome and was discharged from follow-up. However, she noticed small lumps along the vertical scar of both breasts. These first appeared 6 months postoperatively. Although she was not unduly concerned, her general practitioner referred her to a local breast unit where she underwent ultrasound imaging and was reassured that the lumps were benign (Figure 1; 1A,1B). Over the next six years, the lumps continued to slowly increase in size. When they became readily visible through her clothes, she underwent a further assessment in the local breast unit. An ultrasound and mammogram examination (Figure 1) reported benign masses. A core biopsy of one of the masses was performed and it yielded thick sebaceous type material and subsequent histology confirmed it to be consistent with material from an epidermal inclusion cysts. The site of the right core biopsy was slow to heal and resulted in a referral to our plastic surgery department.

**Figure 2.**
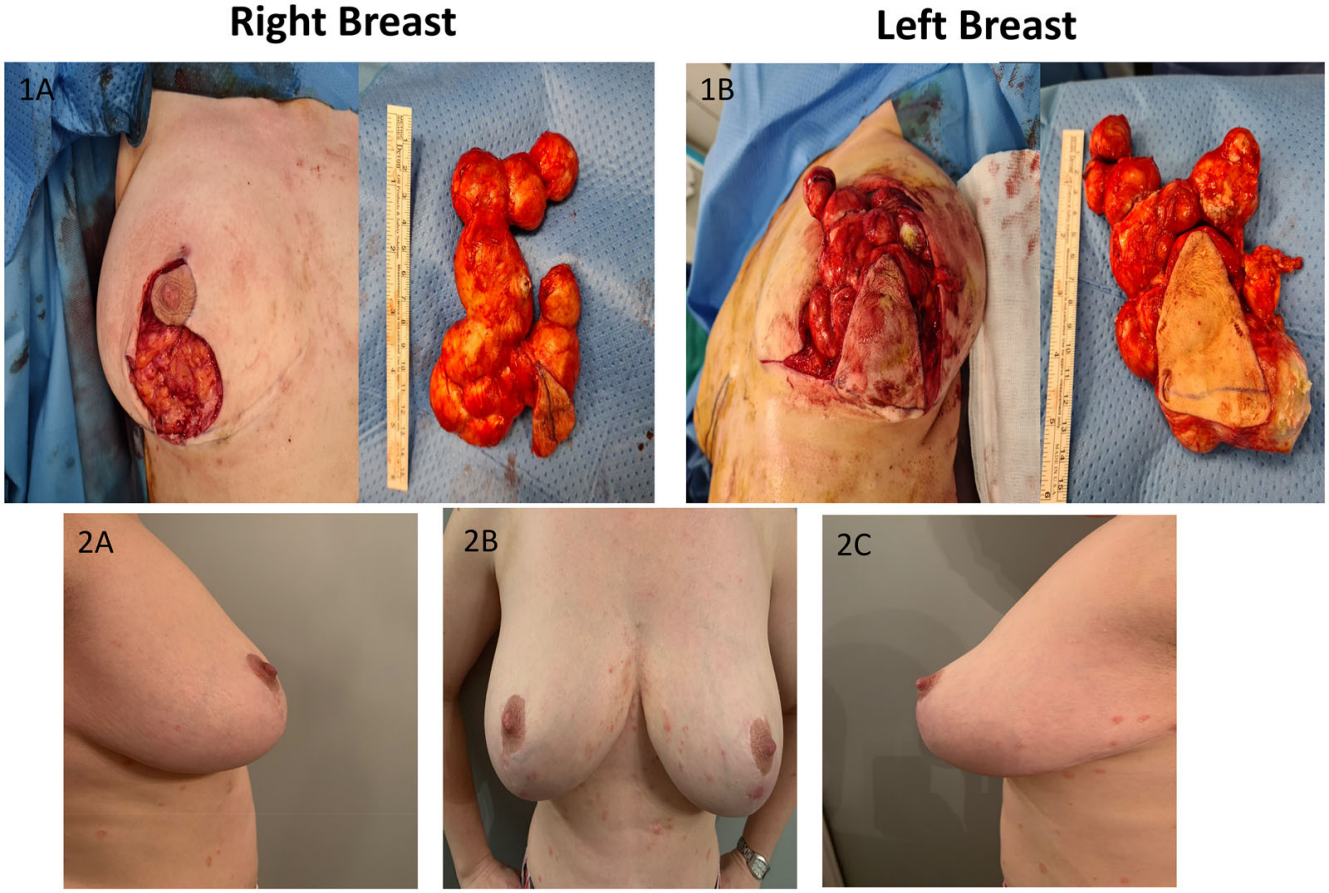
Images of specimens taken intraoperative (1A,1B) and postoperative pictures at 12-month follow-up (2A,2B,2C).

Clinical examination revealed bilateral multi-cystic subcutaneous masses directly under the inferior peri-areolar scars (nipple areolar complex was not involved), along the vertical limbs of the wise pattern scars (this area corresponded to midline of the previously de-epitheliazed inferior pedicle, in which the superior extend was at the inferior NAC margin). There were no other breast masses or palpable axillary lymphadenopathy. We proceeded with excision under general anesthesia by excising the previous vertical limb scars. Extreme care was taken to dissect these subcutaneous masses en-block and the peri-areolar portion was accessed taking care to limit the length of the incision (Figure 2). No additional skin or breast tissue resection performed after en-block resection of the cysts. Closure of wound was identical to the first surgery. However, in this procedure, she received intraoperative antibiotic (co-amoxiclav) and per oral dose for 1 week. Histology confirmed multiple epidermal inclusion cysts ranging from 1.5 to 5.5 cm cysts, with focal rupture, foreign body giant cell reaction and no evidence of malignancy. As a result of precise surgical planning, there were no compromise to NAC and wound healing. The patient was doing well at 12 months postoperative with repeat mammogram revealed no residual cysts and pleasing cosmetic outcome.

(Figure 2; 2A,2B,2C-Of note, no pre-operative or previous medical photography available for comparison). She was diagnosed with mild psoriasis (the rash is noticeable on Figure 2B) after the surgery and treated with topical therapy.

## Discussion

Epidermal inclusion cyst (EIC) of the breast is a naturally slow-growing and rare condition. To date, 100 cases of EIC were reported in the literature from numerous case reports and one literature review (Table 1) [2–7,9–15]. Clinical presentation varies. It ranges from the more commonly palpable mass to bloody nipple discharge or rapidly growing breast mass, which can mimic a malignant neoplasm [3,9,10]. Ultrasound imaging is the most commonly used modality for diagnostic imaging. The typical finding of an ‘onion-ring’ appearance, with alternating concentric hyperechoic and hypoechoic rings in the ultrasound imaging can consistently achieve an accurate diagnosis [3,16,17]. However, in practice, mammogram and magnetic resonance imaging are also used, primarily to rule out malignancy [13,17]. Fine needle aspiration cytology and biopsy are also used as diagnostic tools, however, they are unnecessary if ultrasound imaging is conclusive [3]. Even though EIC is mostly a benign growth, a malignant transformation has been reported, with an incidence between 0.011-19% [14,15]. A literature review of 35 publications on this topic by Paliotta et al. concluded that in cases where the patient is asymptomatic, has a small lesion (<2cm) and an accurate diagnosis with ultrasound imaging, then treatment is not warranted. However, when the EIC is large, palpable and causing patient discomfort, surgical excision is advised with the removal of an intact cyst wall for pathological analysis. No recurrence of EIC was reported at up to 62 months of follow-up [3]. Concerning EIC after reduction mammaplasty, it has been reported to occur as early as three weeks postoperative (ranges from three weeks to eight years) and after excision of EIC, the longest follow-up was two years and revealed no recurrence [4,5,7,11,12].

Previous history of trauma or surgery to the breast (such as needle biopsy, excision, reduction mammaplasty and breast reconstruction with latissimus dorsi) has been reported to be associated with this benign tumor [4–6,10,11]. It is postulated to be caused by epidermis, either that has been left when de-epithelializing the skin, or implanted within the breast tissue, which was then buried. In a mammaplasty procedure, the nipple areolar complex is repositioned along with a vascularized tissue pedicle, requiring in folding of the tissue. Small fragments of epidermis may possibly remain and later result in the development of epidermal inclusion cysts on the medial or lateral skin flaps along the inframammary incision line [3,10,11]. Although breast EIC after mammaplasty have been described, nonetheless, all cases have been unilateral [3,6,11,12]. This is the first case report where EIC presented bilaterally. The patient had a diagnosis of mild hidradenitis suppurativa and it is not clear if this contributed to her presentation [18]. She has no other history of epidermal inclusion cyst in any other location, dental anomalies, gastrointestinal symptoms or family history of colon cancer, making association with Gardner’s Syndrome unlikely [19].

## Conclusion

Epidermal inclusion cyst (EIC) of the breast after reduction mammaplasty is rare. However, it can be quite disfiguring when it becomes enlarged. Ultrasound and clinical examination are usually diagnostic. Excision using existing scar lines is possible and safe, even in cases where the cyst are multiple and up to 5.5 cm in diameter. From surgical perspective, meticulous complete de-epithelialization of the skin during reduction mammaplasty is important to avoid an EIC occurrence.

## Consent form

Informed consent from the patient has been obtained for usage of her clinical details and clinical photographs for publication.

## Figures and Tables

**Table 1.** Previous reports of epidermal inclusion cyst of the breast.

Authors	Published year	Journal title	Number of cases	Trauma history/surgery	Largest size / breast involvement
Hernández-Villafranca at al. [9]	2021	The Breast Journal	1	No	2.2 cm Unilateral breast
Kim et al. [10]	2019	The American journal of case reports	1	No	1.8 cm Unilateral breast
Paliotta et al. [3]	2016	Oncology Letters	82	18 cases had previous cosmetic surgery/trauma	10.0 cm Unilateral breast
Wynne et al. [15]	2015	Radiology case reports	1	Reduction mammaplasty	1.7 cm Unilateral breast
Schaverien et al. [5]	2014	The Breast Journal	1	Mastectomy and immediate latissimus dorsi reconstruction	3.7 cm Unilateral breast
Chopra et al. [11]	2012	Eplasty	5	Reduction mammaplasty	Not available
Singh et al. [2]	2012	Journal of Cytology	6	No	1.5 cm Unilateral breast
Suhani et al. [16]	2011	Breast Disease	1	No	5.0 cm Unilateral breast
Mu et al. [12]	2010	Annals of Plastic Surgery	4	Reduction mammaplasty or Mastopexy	Not available
Herreros-Villaraviz et al. [4]	2008	The Breast Journal	1	Reduction mammaplasty	2.0 cm Unilateral breast
Davies et al. [6]	1997	Histopathology	5	Not available	Not available
Fajardo et al. [7]	1993	Radiology	3	Reduction mammaplasty	1.6 cm Unilateral breast
Menville [17]	1936	Annals of Surgery	36	Not available	Size not available Unilateral breast
